# Stabilizing Mechanisms of β-Lactoglobulin in Amorphous Solid Dispersions of Indomethacin

**DOI:** 10.1021/acs.molpharmaceut.2c00397

**Published:** 2022-09-22

**Authors:** Aleksei Kabedev, Xuezhi Zhuo, Donglei Leng, Vito Foderà, Min Zhao, Per Larsson, Christel A. S. Bergström, Korbinian Löbmann

**Affiliations:** † Department of Pharmacy, Uppsala University, 75123 Uppsala, Sweden; ‡ Department of Pharmacy, University of Copenhagen, 2100 Copenhagen, Denmark; § Zerion Pharma A/S, Blokken 11, 3460 Birkerød, Denmark; ∥ School of Pharmacy, Queen’s University Belfast, Belfast BT9 7BL, U.K.; ⊥ Queen’s University Belfast Joint College (CQC), China Medical University, Shenyang 110000, China

**Keywords:** amorphous solid dispersion, β-lactoglobulin, molecular dynamics simulation, stability, hydrogen bonds, mobility, poorly soluble drugs

## Abstract

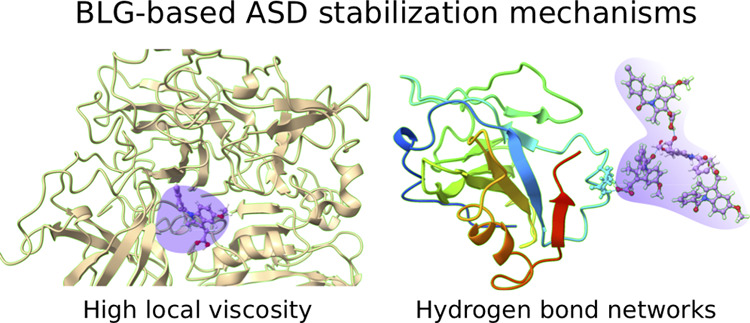

Proteins, and in particular whey proteins, have recently been introduced as a promising excipient class for stabilizing amorphous solid dispersions. However, despite the efficacy of the approach, the molecular mechanisms behind the stabilization of the drug in the amorphous form are not yet understood. To investigate these, we used experimental and computational techniques to study the impact of drug loading on the stability of protein-stabilized amorphous formulations. β-Lactoglobulin, a major component of whey, was chosen as a model protein and indomethacin as a model drug. Samples, prepared by either ball milling or spray drying, formed single-phase amorphous solid dispersions with one glass transition temperature at drug loadings lower than 40–50%; however, a second glass transition temperature appeared at drug loadings higher than 40–50%. Using molecular dynamics simulations, we found that a drug-rich phase occurred at a loading of 40–50% and higher, in agreement with the experimental data. The simulations revealed that the mechanisms of the indomethacin stabilization by β-lactoglobulin were a combination of (a) reduced mobility of the drug molecules in the first drug shell and (b) hydrogen-bond networks. These networks, formed mostly by glutamic and aspartic acids, are situated at the β-lactoglobulin surface, and dependent on the drug loading (>40%), propagated into the second and subsequent drug layers. The simulations indicate that the reduced mobility dominates at low (<40%) drug loadings, whereas hydrogen-bond networks dominate at loadings up to 75%. The computer simulation results agreed with the experimental physical stability data, which showed a significant stabilization effect up to a drug fraction of 70% under dry storage. However, under humid conditions, stabilization was only sufficient for drug loadings up to 50%, confirming the detrimental effect of humidity on the stability of protein-stabilized amorphous formulations.

## 1 Introduction

The amorphous form of a drug has higher apparent solubility and a faster dissolution rate than its crystalline counterpart, which makes it an attractive formulation for poorly soluble drugs.^1^ However, the amorphous form is thermodynamically unstable and usually recrystallizes with time, leading to reduced solubility and pharmaceutical effect of the drug.^2^ To prevent recrystallization, one can stabilize the amorphous form with different excipients that incorporate the drug. This generates a system referred to as an amorphous solid dispersion (ASD).^3^ Numerous formulations are based on synthetic polymers as carriers. In theory, a polymeric ASD can be thermodynamically stable when the drug loading is below the saturation solubility of the drug in the polymer at a given temperature and humidity. However, since the saturation solubility is often far below 30 wt %,^4^ the feasibility of polymeric ASD systems is limited by their low drug loadings, i.e., the amount of drug that can be efficiently incorporated into them.

Recently, interest in the use of proteins as stabilizing excipients for ASD systems has increased, in particular for gelatin, bovine serum albumin (BSA), and whey protein isolate (WPI). For example, gelatin-based ASDs prepared at relatively low drug loadings (<20–30 wt %) show significant improvement in the dissolution rate compared to the crystalline drug.^5^ In another study, gelatin and BSA stabilized the supersaturated state of 12 model drugs in solution, as well or even better than, the synthetic polymers poly(vinylpyrrolidone)/vinyl acetate (PVP/VA) and hydroxypropyl methylcellulose (HPMC).^6^ This could be related to strong interactions between the drugs and proteins in solution.^7^ In particular BSA interacted with all drugs, whereas gelatin only showed interactions with 5 out of the 12 model drugs. This was explained by the globular structure of BSA with several binding sites for the drugs compared to the fibrous (nonglobular) gelatin. While drug loadings for gelatin and BSA were similar to those obtained when using synthetic polymers, it has been suggested that WPI is an efficient excipient in amorphous stabilization, dissolution, and solubility enhancement achieving drug loadings of 50 wt % and higher.^8,9^ Herein, whey proteins were reported to stabilize amorphous indomethacin, furosemide, and carvedilol for at least 27, 17, and 8 months, respectively. WPI is a protein mixture consisting mainly (up to 92% in WPI) of the proteins α-lactalbumin and β-lactoglobulin (BLG), with the latter being the main component (up to 75%).^8^ Hence, the main contribution to the amorphous stabilization, dissolution, and solubility enhancement has been ascribed to BLG.^10^ BLG is a small (18.3 kDa, 162 amino acids), soluble, and globular protein with two disulfide bridges.^11^ It has also been shown that BLG can bind to small hydrophobic ligands, hence, acting as a carrier system of these compounds in solution,^12^ which may additionally be favorable for, among others, supersaturating ASD systems.

However, it is yet challenging to understand the specific factors contributing substantially to the efficient stabilization of ASDs. Experimental techniques often cannot approach the resolution at a scale of nanometers or nano- and micro-seconds. Computer simulations, and molecular dynamics (MD) in particular, can then serve as a useful tool to provide molecular-level information about drug–excipient interactions.^13,14^ Wide spectra of MD simulations, ranging from all-atom resolution and up to significantly coarse-grained models, allows for studying miscibility, mobility, solubility, hydrogen-bond formation, effect of surfactants, and dynamics of the entire system.^15−17^ Such knowledge can then facilitate optimization of the current ASDs and development of new formulations. Among other advantages of MD, there is an ability to a better system control in the simulations than during the experiment, including moisture, temperature, and other factors that could have an effect on the experiment.

In this study, we explored the potential of proteins as amorphous stabilizers using a combination of experimental techniques and computer simulations. The aim was to investigate the key factors determining the stability of the drug molecules around BLG in ASD and to find the optimal drug loading in such formulations for a chosen active pharmaceutical ingredient (API). To target this aim, we prepared the model drug indomethacin (IND) with BLG at different drug loadings. ASD systems with a drug loading in 10% increment steps were prepared by vibrational ball milling and spray drying, and their solid-state characteristics were analyzed using X-ray powder diffraction (XRPD) and modulated differential scanning calorimetry (mDSC). In the molecular dynamics (MD) simulations, we analyzed the formation and longevity of hydrogen bonds in several drug shells around the proteins, the spatial distribution of IND molecules, and their diffusivity at various drug loadings. Taken together, the analytical techniques in this study validated the computational model, whereas the latter provided insights into the molecular mechanisms behind efficient ASD stabilization of IND by BLG.

## 2 Materials and Methods

### 2.1 Materials

Indomethacin (IND; *M*_w_ = 381.37 g/mol; purity 99.1%) was purchased from Fagron (Barsbüttel, Germany). Lacprodan BLG Pharma Grade and β-lactoglobulin (BLG; purity ≥92%) were received from Arla Food Ingredients (Viby, Denmark). Ethanol (95%) was purchased from VWR International (Fontenay-sous-Bois, France) and acetic acid (≥99,7%) from Mallinckrodt Baker B.V (Deventer, The Netherlands).

### 2.2 Methods

#### 2.2.1 Preparation of IND-BLG ASDs by Ball Milling

Briefly, 1000 mg mixtures of IND and BLG at different ratios (from 10:90 to 90:10 in 10% increments) were placed inside 25 mL milling jars with two 12 mm stainless steel balls. Milling was performed continuously for 60 min at 30 Hz and 4 °C with a vibrational ball mill (Mixer Mill MM400, Retsch GmbH & Co., Haan, Germany).

#### 2.2.2 Preparation of IND-BLG ASDs by Spray Drying

IND and BLG were dissolved separately in 190 mL of ethanol and 10 mL of acetic acid, respectively, and then subsequently mixed together to obtain a 200 mL solution (final concentration 5% acetic acid). The solid content dissolved corresponded to a total of 1000 mg of IND and BLG (from 10:90 to 70:30 in 10% increments). After stirring for 20 min, the solutions were spray-dried using a Büchi B-290 spray-dryer (Büchi Labortechnik AG, Flawil, Switzerland) equipped with an inert loop B-295 (Büchi Labortechnik AG). Spray drying conditions were: inlet temperature, 100 °C; outlet temperature, 53–58 °C; feed rate, 10 mL/min; atomization air flow rate, 473 L/h; drying air flow rate, ca. 35 m^3^/h.

#### 2.2.3 Volatile Content Determination

The volatile content of the freshly prepared formulations (moisture or residual solvents) was determined using a Discovery thermogravimetric analyzer TGA (TA instruments, New Castle, DE). The sample was heated from 10 to 300 °C at a heating rate of 10 °C/min, and the volatile content was determined as weight loss between 25 and 150 °C (*n* = 1).

#### 2.2.4 Differential Scanning Calorimetry

The glass transition temperatures (*T*_g_s) of the freshly prepared formulations were determined by differential scanning calorimetry on a Discovery DSC (TA instruments, New Castle, DE). Approximately 9–11 mg of the sample was placed in an aluminum Tzero pan with a perforated hermetic lid. The samples were exposed to a heat–cool–heat cycle using modulated DSC. The ball-milled samples were first annealed at 100 or at 125 °C if spray-dried and then kept isothermal for 10 min to remove any residual moisture/solvent before being cooled to 20 °C. Subsequently, they were heated to 220 °C at a heating rate of 3 °C/min with a modulated temperature amplitude of 1.5 °C and a period of 60 s (*n* = 3).

#### 2.2.5 X-ray Powder Diffraction

XRPD was used to investigate the solid-state characteristics of the samples. The diffraction patterns of samples were recorded using an X’Pert PANanalytical PRO X-ray diffractometer (PANanalytical, Almelo, The Netherlands) with Cu Kα radiation at 1.54187 Å, 40 mA current, and 45 kV acceleration voltage over 5–30° 2θ, at a scan rate of 0.067° 2θ/s, and a step size of 0.026°. The diffraction data were analyzed using X’Pert Data Viewer (version 1.2) software.

#### 2.2.6 Physical Stability

IND-BLG ASD formulations were stored in desiccators at 40 °C/dry (silica gel) or 40 °C/75% relative humidity, achieved over a saturated sodium chloride solution. Periodically, the physical stability of the ASD powders was evaluated by XRPD.

### 2.3 Computational Methods

#### 2.3.1 System Setup

**Figure 1:**
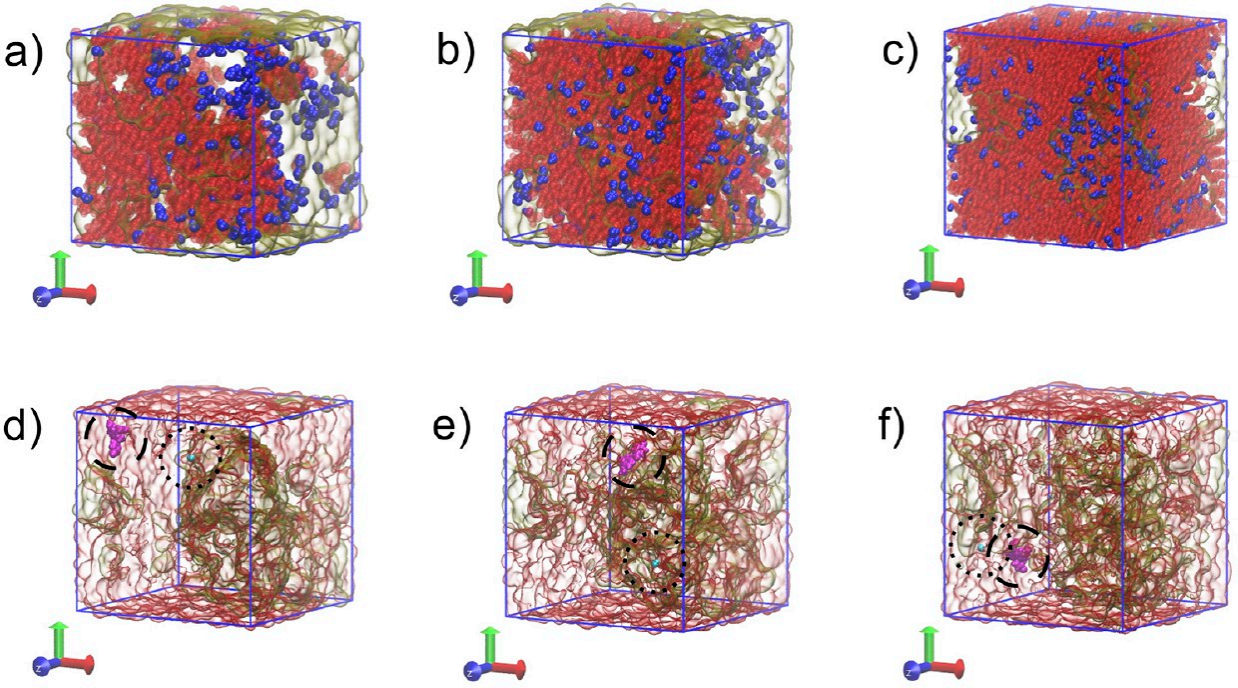
Simulation boxes in humid conditions with (a) 22.5%, (b) 47.5%, and (c) 72.5% drug loadings, as observed at the end of the trajectories. IND, BLG, and water molecules are color coded with red, semitransparent yellow, and blue, respectively. (d–f) Demonstration of the annealing stage in action. A randomly chosen IND molecule and water molecule are depicted with an interval of 10 ns during the annealing stage (550 K). IND is shown in magenta and emphasized with a dashed line circle. The water molecule is depicted in cyan and emphasized with a dotted circle.

MD simulations were conducted on systems with (humid) and without (dry) water molecules at various drug loadings (see examples in Figure 1a–c and Table 1). The humid simulations were run assuming a 5% water content, as an entirely dry amorphous system is not achievable experimentally due to the hygroscopicity of the amorphous materials. In fact, the residual moisture of all formulations was tested and lies within the range of 1.19–7.64% (see Table S1 in the supplementary information). Four or eight individual BLG molecules were introduced in systems with high (>40% API) and low (<40% API) drug loadings, respectively. This was done to meet the requirement of the minimum-image convention, as in the systems with a lower drug mass, four BLGs would not be entirely screened from their own periodic images in small boxes. Different numbers of IND molecules were added to split the drug loading range between 0 and 100% into nine even intervals. The dry systems were simulated at drug weight fractions of 13, 25, 38, 50, 62, 75, 87, and 100%, in which the remaining fraction was represented by BLG molecules. In the humid systems, the number of water molecules was adjusted to contribute 5% to the weight of the entire system. Thus, the corresponding drug fractions were 12% (83% BLG, 5% water), 22.5% (72.5% BLG, 5% water), 35% (60% BLG, 5% water), 47.5% (47.5% BLG, 5% water), 60% (35% BLG, 5% water), 72.5% (22.5% BLG, 5% water), and 83% (12% BLG, 5% water). A full list of simulations and the corresponding box sizes and the number of molecules are listed in Table 1.

**Table 1:** Summary of the Simulation Boxes Used in this Study

dry systems	13/87%	25/75%	38/62%	50/50% IND/BLG	62/38%	75/25%	87/13%	100% IND
no. of molecules,	58 IND, 8 BLG	138 IND, 8 BLG	246 IND, 8 BLG	206 IND, 4 BLG	343 IND, 4 BLG	617 IND, 4 BLG	1440 IND, 4 BLG	500 IND
approximate box side size	6 nm	6.3 nm	6.7 nm	5.7 nm	6.4 nm	7.4 nm	9.5 nm	6.3 nm

humid systems	12/83/5%	22.5/72.5/5%	35/60/5%	47.5/47.5/5% IND/BLG/W	60/35/5%	72.5/22.5/5%	83/12/5%	95/5% IND/W
no. of molecules	58 IND, 8 BLG	138 IND, 8 BLG	246 IND, 8 BLG	206 IND, 4 BLG	343 IND, 4 BLG	617 IND, 4 BLG	1440 IND, 4 BLG	475 IND
approximate box side size	246 W, 6.1 nm	315 W, 6.4 nm	372 W, 6.7 nm	458 W, 5.9 nm	601 W, 6.4 nm	888 W, 7.4 nm	1440 W, 9.5 nm	497 W, 6.4 nm

To accelerate the compound mixing, the systems were annealed, first by raising the temperature to 550 K, then cooling to 200 K in each case, and subsequent equilibration at room temperature (298 K). This thermal annealing induced the translational movement of the drug molecules (and water in the humid systems) that otherwise tend to remain immobile after random placement in the box. Annealing was performed to minimize the impact of the initial configuration; annealing disorders the molecules and the subsequent rapid cooling traps them in the last observed amorphous form. Increasing the temperature to 550 K deletes the memory of the initial configuration from the system (see Figure 1d–f). Despite the rapid annealing, the proteins remained in their folded structure throughout the entire simulations (for both dry and humid systems). Another validation of the annealing efficiency was the comparison of the data for diffusivity and radial distribution function under two starting conditions. In one of them, all compounds were put into the box randomly at the beginning of the annealing simulations, and in another, the proteins were equilibrated first in a single-phase system, followed by addition of IND and finally by water. No significant difference in the data was observed between the simulations with the two protocols of system preparation. Thus, the annealing phase was sufficient to exclude the impact of the initial configuration and did not introduce artifacts in the subsequent simulations.

#### 2.3.2 Molecular Model Parametrization

The generalized Amber force field (GAFF) was used to run the MD simulations at the all-atom scale.^18^ Software packages Stage^19^ and Modeller^20^ were used to develop the models of indomethacin and β-lactoglobulin. The TIP3P model was used as a water model.^21^

#### 2.3.3 Simulation Parameters

Gromacs version 2018 was used to run the MD simulations.^22^ All initial conformations were made with Packmol.^23^ After random placement of all compounds into the initial box, the steepest descent energy minimization was performed for 4500 steps. This was followed by NVT (Nosé–Hoover thermostat, time constant for coupling τ_t_ = 2 ps) and NPT equilibration stages (Berendsen isotropic pressure coupling, τ_t_ = 5 ps and compressibility of 4.5 × 10^–5^ bar).^24,25^ At the production stage, a v-rescale thermostat was used for temperature coupling and a Parrinello–Rahman barostat for isotropic pressure coupling.^26^ Periodic boundary conditions were applied in all three dimensions. The timestep was set to 2 fs for production simulations and 200 ns simulations were run in duplicate for each system. Of the 200 ns simulations of the systems with high drug loading, 10 ns was spent increasing the temperature from 298 to 550 K, 90 ns for mixing at 550 K, followed by cooling down to 200 K for 20 ns, then increasing the temperature to 298 over 20 ns, and the final 60 ns at room temperature (see the temperature profile in Figure S1). For the low drug loading systems, the total simulation time was 1140 ns, with the room temperature stabilized after 140 ns, similar to the smaller scale setup. Then, the last 20 or 900 ns of the production simulations were used for the analysis in the respective cases. The linear constraint solver (LINCS) algorithm^27^ was used to constrain bonds involving hydrogen atoms, and particle mesh Ewald summation^28^ was used for electrostatic interactions. Cutoff distances for both Lennard-Jones and electrostatic interactions (short range) were set to 1.2 nm.

#### 2.3.4 Analysis

We analyzed the data using Gromacs built-in tools,^22^ VMD software,^29^ and in-house scripts. gmx rdf was used to analyze the likelihood of the molecules being at certain distances from each other. We applied “gmx hbonds” (with a cutoff angle of 30° and cutoff distance of 0.35 nm) to count the number of hydrogen bonds and to create the hydrogen-bond existence matrices and corresponding index files. Additional stability analysis of hydrogen bonds over the last 20 ns of the simulation was done using the script readHBmap.py.^30^ Hydrogen bonds were considered stable if they existed in more than 80 and 50% of the analyzed simulation frames for BLG-IND and IND-IND/BLG-BLG, respectively. We evaluated the minimal distances between the protein and IND molecules with the “gmx mindist” tool. “gmx msd” with the “-mol” option was applied to measure the diffusivity of the individual IND molecules and then to estimate the average diffusivities of specific groups. Lennard-Jones and electrostatic interactions were analyzed with “gmx energy”.

## 3 Results and Discussion

### 3.1 ASD Characterization, Physical Stability, and MD Model Validation

As the first step of the study, we performed experiments and validated the MD model. In Section 3.2, the validated model is used to study specific molecular mechanisms that contributed to improving the stability of the ASDs.

#### 3.1.1 Preparation and Solid-State Characterization

Directly after preparation, the ball-milled and spray-dried formulations were analyzed by XRPD to confirm the amorphicity. All formulations showed the characteristic amorphous halo (Figure S2). IND-BLG ASDs were amorphous regardless of the preparation technique or blend ratio (ball milling: IND-BLG ratio is between 10:90 and 90:10; spray drying: IND-BLG ratio is between 10:90 and 70:30).

The samples were subsequently analyzed with mDSC to study whether the formulations were homogeneous amorphous single-phase systems or amorphous multiphase ones. Table 2 summarizes the thermal analysis. Ball-milled formulations showed a single *T*_g_ for the drug loadings of 10–50%, indicating that they were homogeneous amorphous single-phase systems. In contrast, the formulations with a drug loading of above 50% showed a second *T*_g_, suggesting that these were heterogeneous amorphous mixtures. Findings for all spray-dried formulations were similar; however, only formulations up to a drug loading of 40% were homogeneous amorphous single-phase systems with a single *T*_g_. Furthermore, apart from the 50% drug-loaded formulation, *T*_g_ values for the two manufacturing techniques were similar, suggesting that the formulations are similar despite the different preparation techniques.

**Table 2:** *T*_g_ Values of ASDs Prepared by Ball Milling and Spray Drying for the Investigated Drug Loading (DL) Values^a^

	feed (mg)		ball milling		spray drying	
DL (wt %)	IND	BLG	*T*_g_1__ (K)	*T*_g_2__ (K)	*T*_g_1__ (K)	*T*_g_2__ (K)
90	900	100	334.9 ± 1.9	406.2 ± 1.2		
80	800	200	340.9 ± 1.0	401.4 ± 1.1		
70	700	300	343.3 ± 1.0	406.6 ± 1.2	340.4 ± 3.6	416.4 ± 0.1
60	600	400	351.2 ± 3.3	412.7 ± 3.6	344.7 ± 1.9	412.9 ± 2.2
50	500	500	378.0 ± 2.2		360.4 ± 0.9	423.6 ± 1.4
40	400	600	399.2 ± 0.5		391.2 ± 1.8	
30	300	700	420.8 ± 0.7		424.8 ± 3.3	
20	200	800	445.4 ± 2.6		444.9 ± 1.2	
10	100	900	465.4 ± 2.3		462.4 ± 0.6	

a Data is presented as an average of triplicates ± standard deviation.

**Figure 2:**
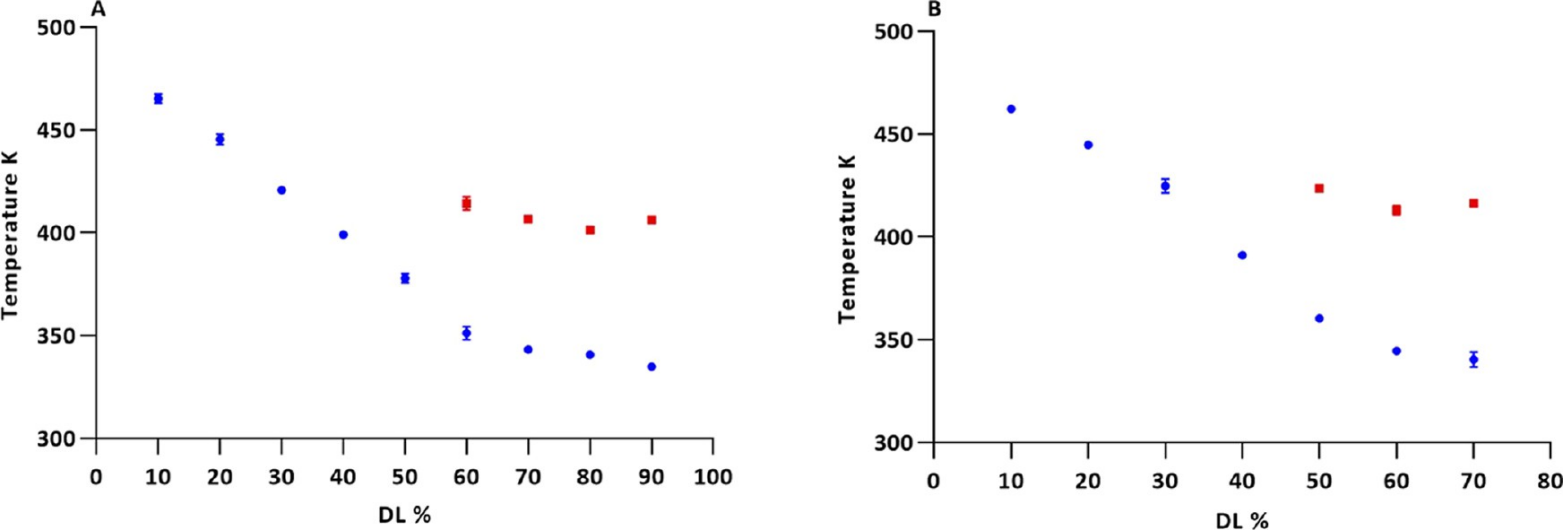
Experimentally obtained *T*_g_ values as a function of drug loading (DL). (A) Ball-milled IND-BLG ASDs and (B) spray-dried IND-BLG ASDs; all values in triplicate.

The appearance of a single *T*_g_ below and two *T*_g_s above a certain drug loading suggests that there are limitations in obtaining a homogeneous amorphous mixture of the IND with BLG. This can be the result of either limited miscibility of IND with BLG or that the binding sites on the BLG surface become saturated with IND molecules at a particular drug loading. When two *T*_g_s were obtained, the lower one (*T*_g_1__) could be attributed to a drug-rich amorphous phase (or possibly a pure amorphous drug; see MD simulations in Section 3.2). The higher *T*_g_ (*T*_g_2__) can be assigned to a BLG-rich amorphous phase, or a drug-saturated IND-BLG mixture (see Section 3.2). *T*_g_ values are plotted in Figure 2. Lower *T*_g_s (*T*_g_1__) values decrease with increasing-IND loading, while the higher ones (*T*_g_2__) for the ball-milled samples were similar to each other (406.7 ± 4.6 K). For the spray-dried samples, the *T*_g_2__ values were likewise similar to each other (417.6 ± 5.5 K).

On the basis of these findings, it seems that the addition of IND up to the threshold of 40–50% leads to formation of homogeneous amorphous single-phase systems with a single *T*_g_, whereas drug loadings above this threshold lead to formation of a measurable drug-rich (or drug-only) clusters corresponding to a *T*_g_1__ event.

#### 3.1.2 BLG-IND Distances Studied Computationally

**Figure 3:**
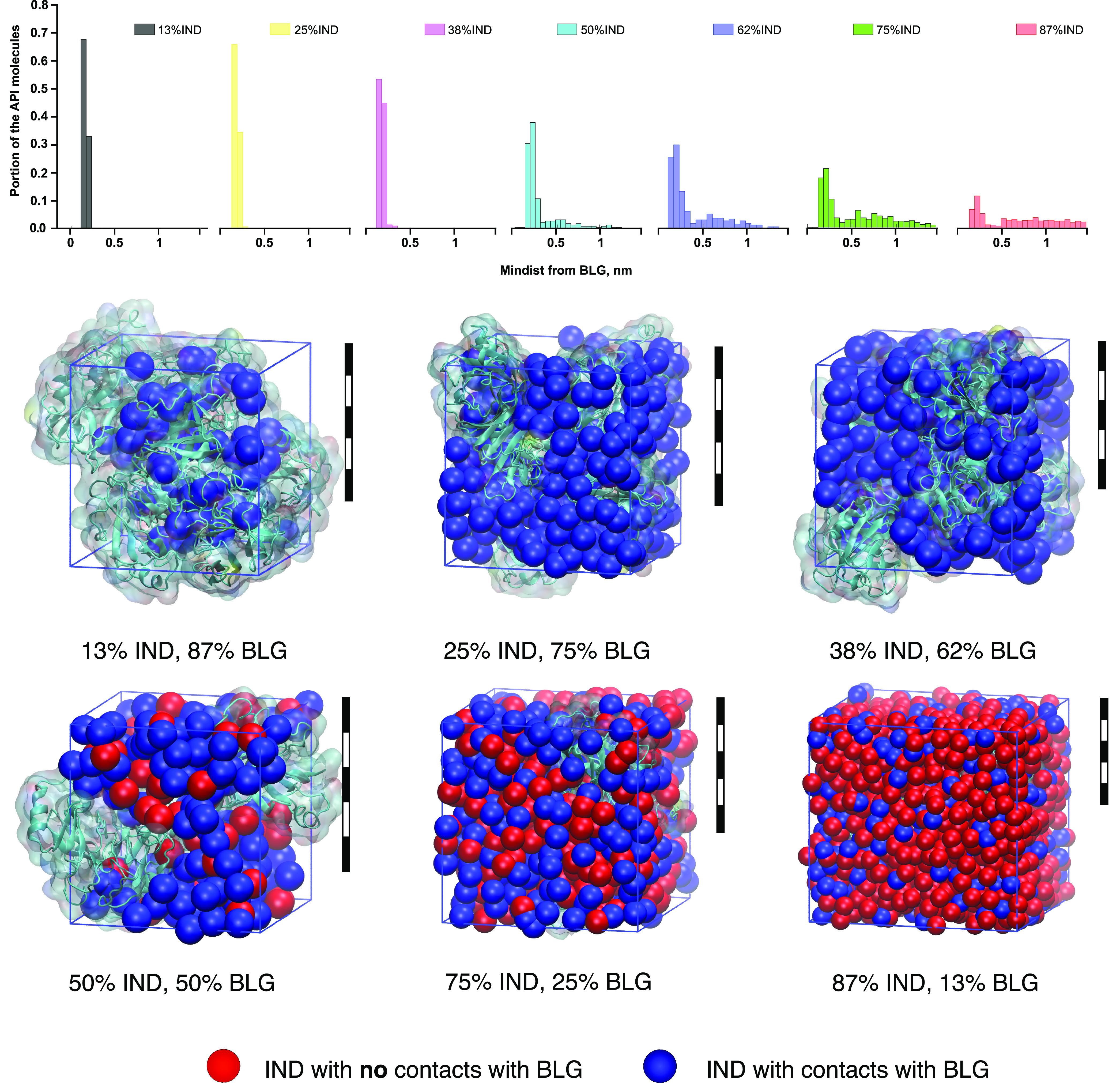
Distributions of the minimal distances between the indomethacin (IND) molecules and the surface of the β-lactoglobulin (BLG) molecules. Top panel: histograms of minimal distances between the surface of the protein and IND center of mass for dry systems (histograms for humid systems are presented in Figure S3). The second peak is detectable at 50% loading but is significantly more pronounced at higher loadings. Middle and lower rows: spatial distribution of the APIs that do (blue) and do not (red) have direct contact with BLGs (turquoise). The second drug layer starts to form in the range between 38 and 50% drug loading. Scale bars stand for 5 nm in all subpanels.

To evaluate the interactions between IND and BLG as well as the formation of IND-rich clusters (or layers) around BLGs, we analyzed the protein–drug simulations and measured the minimal distances between the individual IND molecules and the surface of the BLG molecules after thermal annealing. The shortest distance between any atom of the BLG proteins and the center of mass of APIs was measured throughout the final 20 ns of the simulations. These distances were plotted in a histogram for the different drug loadings (Figure 3, top panel, shown only for dry systems; data for the humid systems is presented in Figure S3). The radius of gyration of the IND molecule around the minor axis (lowest average distance from the center of the molecule to its surface) is approximately 0.22 nm. As can be seen, at 38% drug loading, the entire set of IND molecules is located within a compatible distance, i.e., at the very surface of the protein. Thus, at drug loadings of 38% and below, the IND molecules do not form a drug-rich phase (or the 2nd layer), as virtually all APIs are in contact with the surface of the proteins. Somewhere between 38 and 50%, some molecules start to appear at farther distances from the surface.

At the next subpanel (Figure 3, 50% API), a bimodal distribution occurs, with the second mode being maximal at about a doubled distance between the first and the BLG surface. We propose that this indicates the beginning of the second drug layer or the formation of IND clusters at a drug fraction of ∼40–50%. By a second drug layer, we mean the drug molecules that are only in contact with other drug molecules. At IND fractions higher than 62%, the distribution gradually levels off, whereas the trend of IND molecules accumulating near the protein surface is still there.

It is important to note here, that it does not necessarily mean a uniform first layer of the IND molecules covering the entire surface of BLGs. The bottom panels of Figure 3 show that the protein surface is not entirely covered at 38% or even 75% drug loading. For these drug loadings, the proteins tended to aggregate (including via the periodic boundary condition, see also Figure S4), and a single BLG cluster was only screened from the appearance of its periodic images at 87% drug loading and above. The blue beads represent the IND molecules in contact with the BLG surface, and the red beads represent those that do not have protein atoms within 0.4 nm from the surface. IND depiction is presented in Figure S4.

Thus, overall our mDSC and MD data are in good agreement with each other. The mDSC data suggested that IND clusters that are not in direct contact with the BLG surface start to form at a drug loading above 40 and 50% for the spray-dried and ball-milled samples, respectively. These represent a drug-rich phase, which is detected as a separate *T*_g_ in the differential scanning calorimetry (DSC) measurements (Figure 2). The MD data also suggested that IND clusters start forming at a drug loading in the range between 38 and 50%. Other techniques, such as solid-state nuclear magnetic resonance (NMR), may in the future be valuable to study the systems in more detail and provide a closer link to the MD data.^31,32^

#### 3.1.3 Physical Stability Assessment

To test whether the appearance of the second phase destabilized the samples, ball-milled and spray-dried ASD formulations were stored under dry and 75% RH conditions at 40 °C.

The data obtained from this accelerated stability study are summarized in Tables 3 and 4. Both the ball-milled and spray-dried IND-BLG ASDs remained stable at loadings of up to 70–80% for at least 12 months under dry conditions. Recrystallization was detected at loadings of 80% and higher when stored at 0%RH and at the loadings between 40 and 60% and higher when stored at 75%RH. The lower stability under humid conditions can be explained by moisture sorption and plasticization of the amorphous mixtures as well as competition for BLG binding sites and replacement of the drug by water molecules, all of which accelerate the recrystallization process.

**Table 3:** Physical Stability of Ball-Milled IND-BLG Samples at 40°C^a^

	1 month		2 months		3 months		4 months		12 months	
DL (wt %)	0% RH	75% RH	0% RH	75% RH	0% RH	75%RH	0% RH	75% RH	0% RH	75%RH
90	**R**	**R**	**R**	**R**	**R**	**R**	**R**	**R**	**R**	**R**
80	**R**	**R**	**R**	**R**	**R**	**R**	**R**	**R**	**R**	**R**
70	A	**R**	A	**R**	A	**R**	A	**R**	A	**R**
60	A	**R**	A	**R**	A	**R**	A	**R**	A	**R**
50	A	A	A	**R**	A	**R**	A	**R**	A	**R**
40	A	A	A	A	A	A	A	**R**	A	**R**
30	A	A	A	A	A	A	A	A	A	A
20	A	A	A	A	A	A	A	A	A	A
10	A	A	A	A	A	A	A	A	A	A

a A: amorphous. **R**: recrystallization. DL: drug loading.

**Table 4:** Physical Stability of Spray-Dried IND-BLG Samples at 40°C

	1 month		2 months		3 months		4 months		12 months	
DL (wt %)	0% RH	75% RH	0% RH	75% RH	0% RH	75% RH	0% RH	75% RH	0% RH	75% RH
70	A	A	A	A	A	**R**	A	**R**	A	**R**
60	A	A	A	A	A	**R**	A	**R**	A	**R**
50	A	A	A	A	A	A	A	A	A	A
40	A	A	A	A	A	A	A	A	A	A
30	A	A	A	A	A	A	A	A	A	A
20	A	A	A	A	A	A	A	A	A	A
10	A	A	A	A	A	A	A	A	A	A

Samples at the drug loadings under 40% remained stable up to the end of the measurements even in the presence of high humidity, which indicates the higher stability of the ASDs for one-phase systems. In general, spray-dried samples were more stable and only recrystallized at loadings higher than 50% under humid conditions. Nevertheless, even at higher drug loadings (up to 70% drug loading), samples remained stable under dry storage conditions.

#### 3.1.4 Diffusivity of the Molecules Studied with Molecular Dynamics

**Figure 4:**
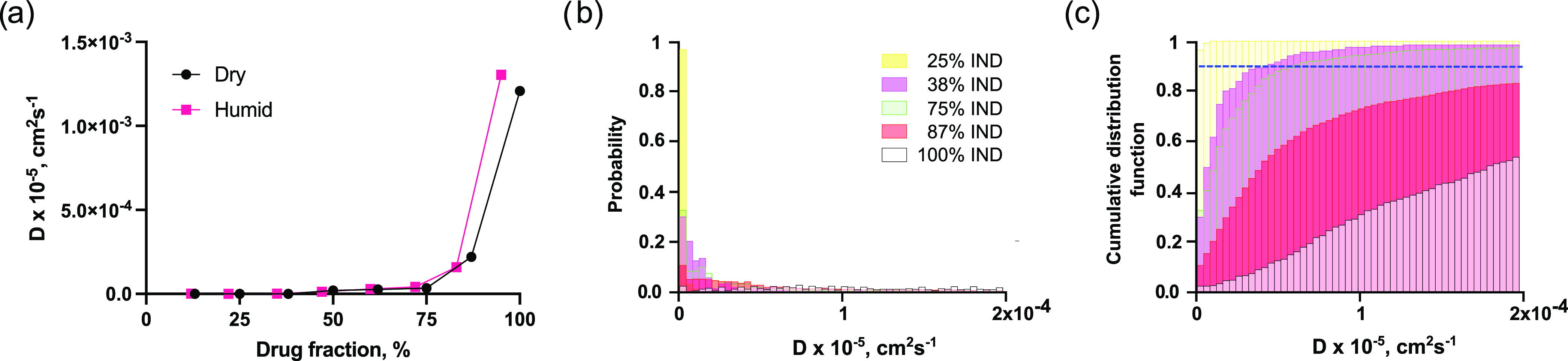
Mobility of the molecules grows dramatically at drug loadings above 75%. (a) Average diffusivity of the indomethacin molecules. (b) Histograms showing the probability of the indomethacin molecules to have a specific diffusivity for several of the studied drug fractions in dry conditions. (c) Cumulative distribution function of the molecules with specific diffusion coefficients into the total diffusivity. The blue dashed line depicts 0.9% of the total diffusivity.

With the results from the physical stability study, we used MD simulations to analyze the average diffusivity (D) of the IND molecules (Figure 4a) and to calculate histograms of the individual diffusivity values at different loadings from the MD simulations and the cumulative distribution functions of the molecules’ diffusivity (Figure 4b,c). As shown in Figure 4a, the average mobility of all molecules differs significantly only at the highest drug fractions of 83% (humid system) and 87%, (dry). In these cases, D is not as great as in the BLG-free systems (100 and 95%, pure IND and “IND and water” systems, respectively). However, D is higher than those of 72 and 75% of drug-loaded ASDs by half an order of magnitude. This suggests that the BLG-stabilization of IND is at least efficient to some extent at the drug fractions of up to 75–80%. In fact, these data correlate very well with the stability study, where formulations at drug loadings of up to 70% remained amorphous under dry conditions (Tables 3 and 4).

In Figure 4b, the distribution of D for individual molecules flattens out with the growth of the drug fraction. At the loadings below 35%, the distribution had a very steep form, as most of the molecules were barely moving in such systems. After passing that point, there was no qualitative difference between the lower drug fraction (38%) and the higher one (75%). A clear flattening of the distribution only started from 87% (Figure 4b). For that fraction, a high number of molecules were almost immobile (same region as the majority of the 25% API system histogram), but the rest had a diverse distribution of diffusivity values. This, together with the minimal distance data (Figure 3), indicates that the placement of IND in the first drug shell (FDS) of BLG is most likely sufficient to stabilize the drug molecules. At the same time, the stabilization effect was not limited to the first shell. Stability was strong enough up to 83% drug loading, as can be seen from the average diffusivity values (Figure 4a). As IND has one hydrogen bond donor and four acceptors, it can form multiple hydrogen bonds with both BLG and other IND molecules further supporting the formation of stable amorphous formulations beyond the first shell of IND bound to BLG. Figure 4c emphasizes this observation: for the system with only 25% IND, the entire mobility of the APIs is reached only by barely moving molecules. For the boxes with 38 and 75% IND, the cumulative distribution functions are more gradual and very similar between themselves, but one can see a shift to the right along the 90% of the total diffusivity level (dashed line in Figure 4c). In other words, molecules with higher diffusivity values (approximately 0.5 × 10^–9^ cm^2^/s) contribute more to the total mobility of the 75% IND system's APIs than those in the 38% IND system. It is also clear from the figure that distribution dramatically changes from 75 to 87%, as in the latter system the most mobile molecules contribute much more to the total diffusivity of the system. In this regard, the distribution recalls the pure API system more than the one with 75% drug loading. It confirms that starting from some point between 75 and 87%, a portion of molecules not stabilized by the BLGs rapidly grows.

All of these observations lead to three possible explanations. (1) The FDS has limited mobility and simultaneously slows down the motions in the subsequent drug layers. Hence, the non-FDS molecules are stabilized due to the FDS slowing down the motion of the closest neighbors (roughly speaking, it induces a higher local viscosity^33^). (2) A hydrogen-bond network originates from the proteins and spans over at least several drug shells around the surface. As can be seen in Figure 3, at a 62% drug fraction, there are molecules located further than 1 nm away from the surface of BLG that still have low diffusivity. (3) The limited mobility of the IND molecules is caused by a combination of both (1) and (2).

Relatively low diffusivity of the molecules next to the BLG surface compared to those in the bulk would validate the first explanation. To test the second (and the third) explanations, we would need to analyze the hydrogen bonds formed by IND with other IND molecules, as well as with BLG and water. The presence of several generations of hydrogen bonds forming a network and their relatively low mobility would indicate the correctness of the second (and, thus, the third) arguments.

### 3.2 Computational Analysis of the Stabilization Mechanisms

In the simulations, diffusivity of the IND molecules increased dramatically at loadings higher than 75%. In the experiments, the ASDs stored under dry conditions remained stable at loadings below 80%. The agreement of these computational and experimental data, taken together with the data in Sections 3.1.1 and 3.1.2, supports the validity of the computational model. Thus, we decided to use MD simulations to investigate additional stabilizing mechanisms. As proposed in the previous section, hydrogen-bond network formation might be one of the key stabilization factors. We, therefore, analyzed the correlation between the hydrogen-bond patterns and mobility of the drug molecules within ASDs.

#### 3.2.1 Hydrogen Bonds and Diffusivity Patterns

**Figure 5:**
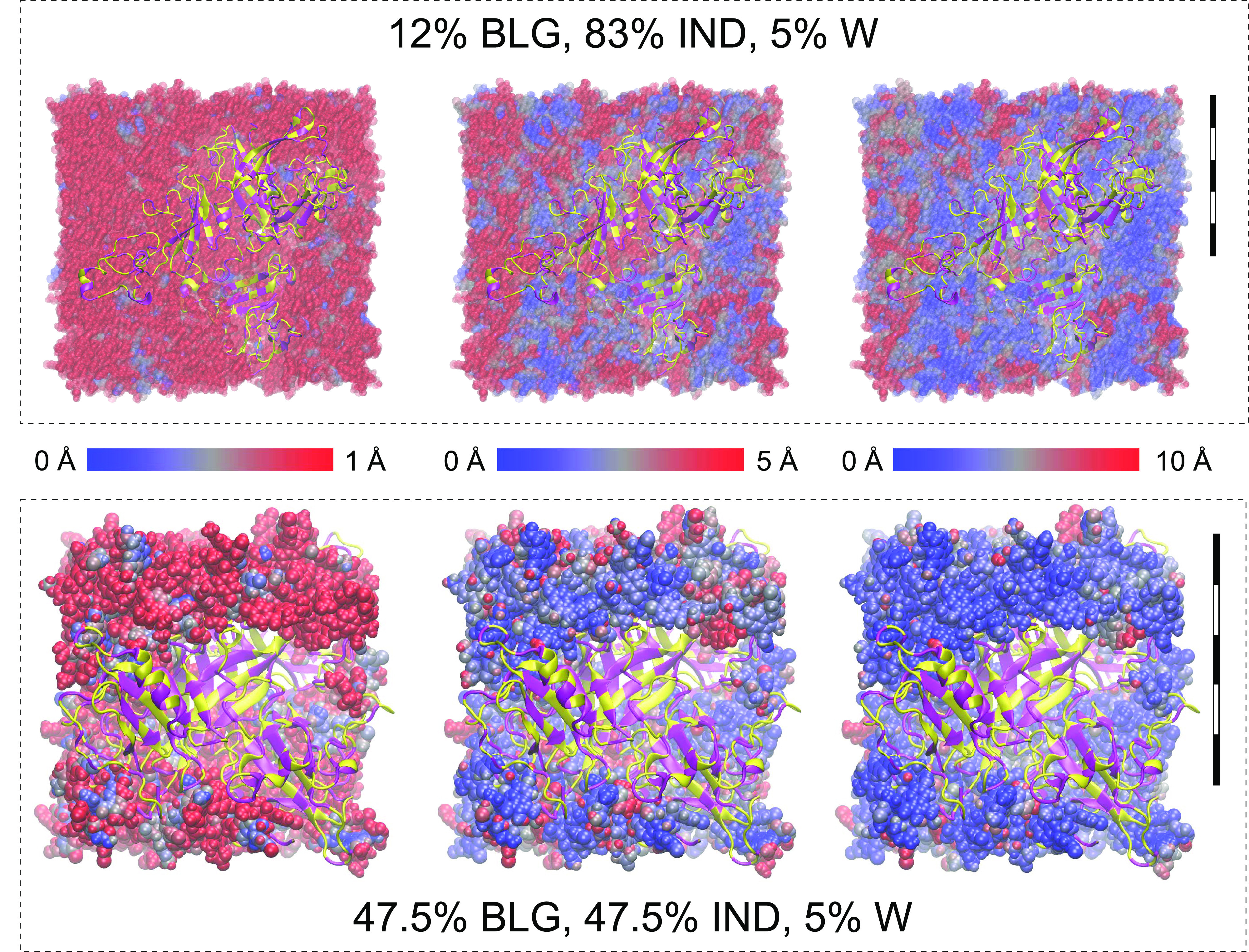
Motion of the indomethacin (IND) molecules in the simulation boxes. The molecules in blue are at the lower end of the spectrum (moving little or not at all) and the red ones move not less than the top value of the range. The proteins (BLG) are depicted with a cartoon representation in two colors: hydrophilic parts of BLG in yellow and hydrophobic in magenta. W: water. Scale bars stand for 5 nm in both subpanels.

First, we visually observed the mobility of the IND molecules around the BLGs. In Figure 5, the API molecules are color coded from blue to red through gray. Red indicates molecules that move as much as, or more than the higher limit of the range (1, 5, or 10 Å, from left to right) within the last 20 ns of the simulation. The protein motion is subtracted from the calculation so that BLG serves as a reference for the measured molecular mobility. The left panel of Figure 5 shows that all IND molecules were mobile to at least a small extent if movement more than 1 Å is considered the highest limit. The central panel shows an intermediate range of displacements from the initial positions. The pattern of the less mobile IND locations is definitely not limited to just the first drug shell. Interestingly, the pattern does not cover the entire surface of the protein. Finally, the right panel shows two categories of molecules. In the first, molecules are relatively immobile (depicted with blue), whereas molecules from the second category move more than 10 Å away from their initial positions in the given time interval.

**Figure 6:**
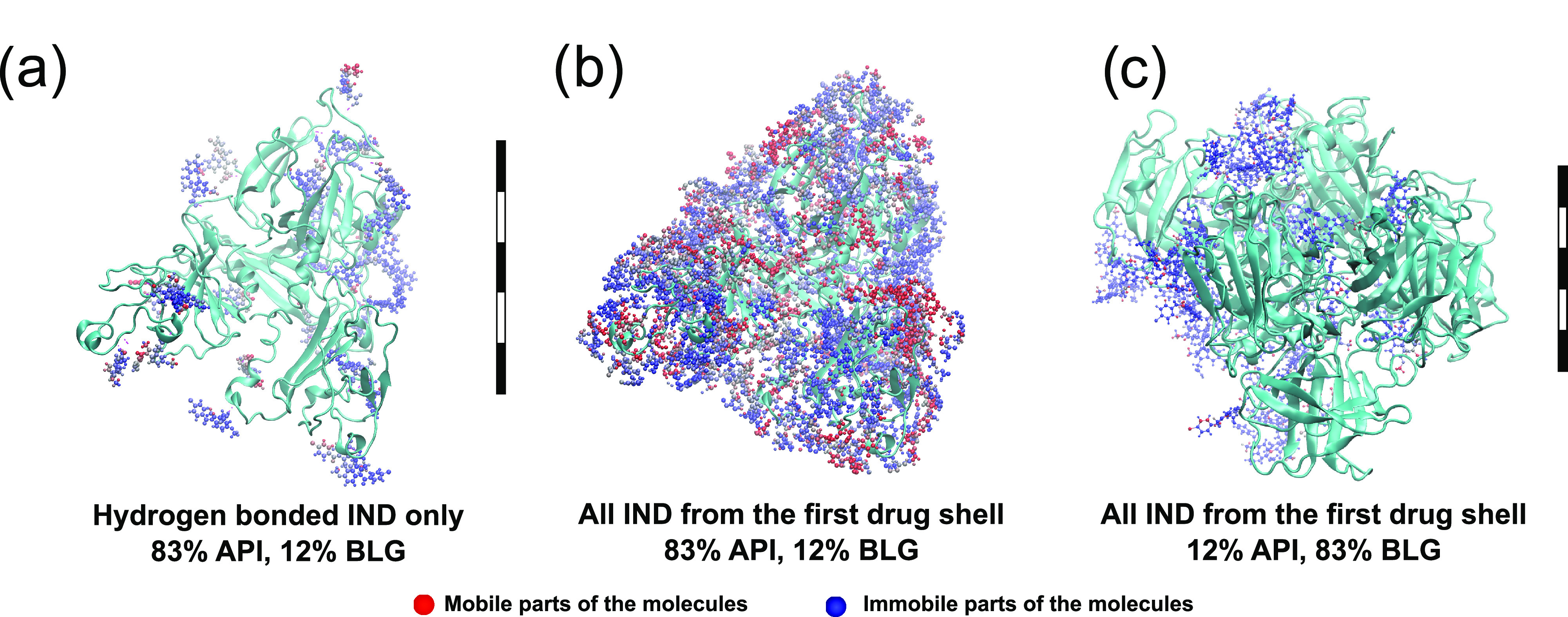
Hydrogen bonds and the mobility of the molecules in the first drug shell. The mobility of the atoms in the last 20 ns of the simulation is color coded: bright blue 0 Å, gray 2.5 Å, and bright red 5 Å. (a) Only hydrogen-bonded IND molecules from the first drug shell are shown on the top of the proteins (humid conditions, 83% drug loading). All bonded molecules remain barely mobile throughout the simulation. (b) All indomethacin (IND) molecules (humid conditions, 83% drug loading) from the first drug shell, including the ones without hydrogen bonding. The figure clearly shows significantly higher average mobility of the nonbonded molecules in the first drug shell; the number of red atoms is increased compared to panel (a). In other words, not all of the molecules from the first drug shell are relatively immobile. (c) All IND molecules (humid conditions, 12% drug loading) from the first drug shell, including ones without hydrogen bonding. There is significantly lower average mobility of all molecules in the first drug shell compared to either panels (a) or (b). Combination of steric hindrance and hydrogen bonds is extremely strong at low drug loadings. Scale bars stand for 5 nm in all subpanels. The left scalebar is valid for panels (a, b), and the right one is for panel (c).

It is noteworthy that some IND molecules located next to the BLG are more mobile than IND molecules placed at further distances from the protein. To confirm this observation with better precision, we analyzed simulations at 83 and 12% drug loadings (Figure 6a–c). We only visualized: (1) the APIs with stable hydrogen bonds to the protein (Figure 6a); and (2) the entire first drug shell, including the IND molecules without hydrogen bonds to BLG (Figure 6b,c). The same color coding is used here for the mobility of the atoms, from 0 Å (bright blue) to 5 Å (bright red). For molecules that were only hydrogen bonded, the major mass of the IND atoms is colored blue. Several atomic groups can be more mobile within the molecule, but this did not seem to affect their center of mass. In the second visualization, many more red-colored molecules were presented, which confirms our hypothesis that even within the FDS, there is differentiation with respect to the mobility of the molecules. However, such differentiation is more relevant to high drug loadings, as otherwise most of the space around each IND would be occupied by immobile BLGs, thus limiting their ability to move long distances. As can be seen in Figure 6c, neither of the molecules was in the red range of mobility in the system with 12% API. The same trend was observed for the molecules of up to 38% IND loading. The appearance of a second *T*_g_ at 40–50% drug loading indicates not only the formation of a drug-rich phase but also the presence of relatively mobile IND molecules.

**Figure 7:**
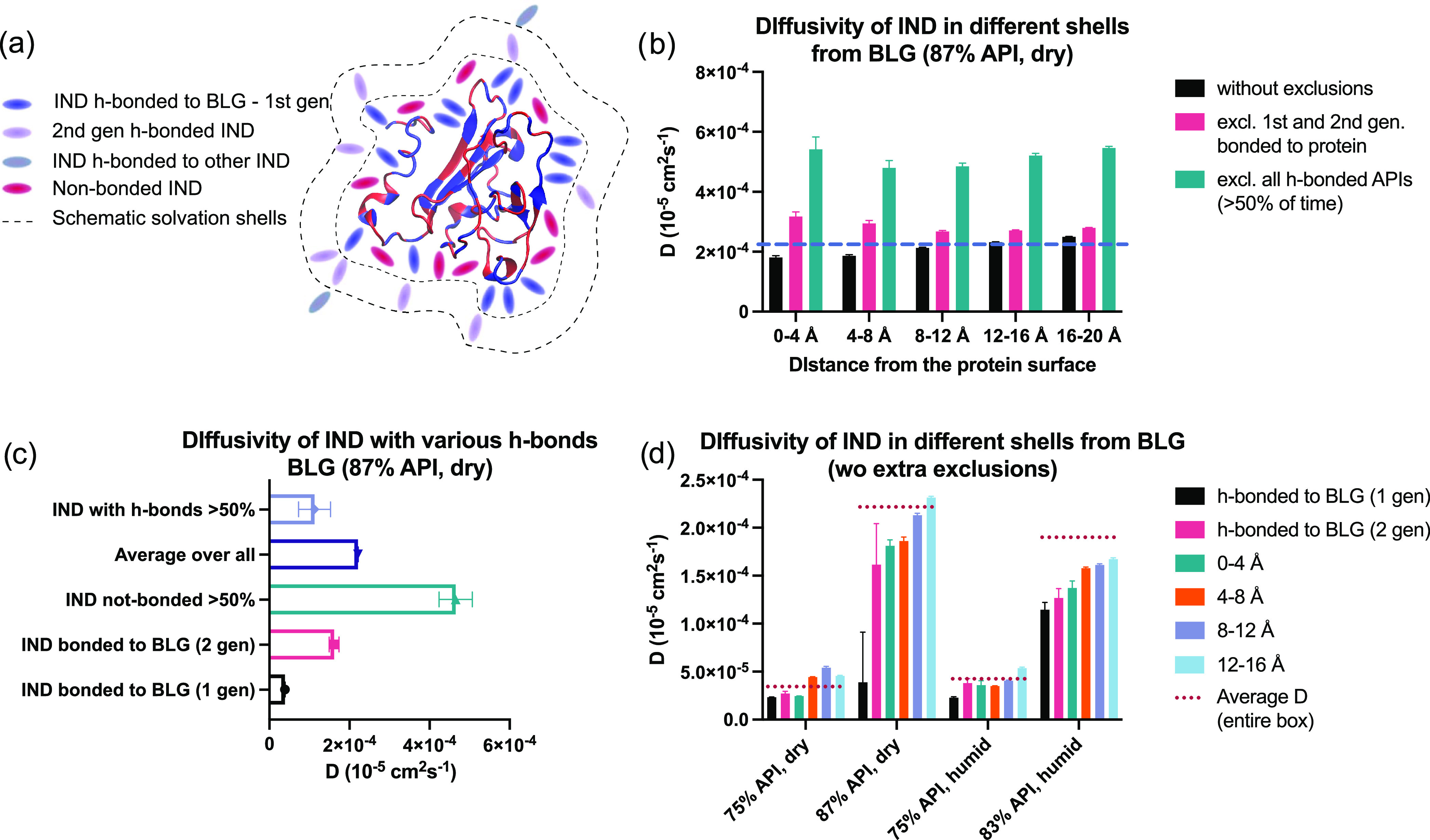
Hydrogen bonds (h-bonds) of the first and the second generations contributed the most to the stabilization of the indomethacin (IND) around β-lactoglobulins (BLGs). However, when all IND molecules with any hydrogen bonds were excluded, the average diffusivity of the drug mass increased significantly. In ASDs with high drug loadings, hydrogen bonds affected API diffusivity more than the distance from the BLG surface. (a) Schematic representation of the drug shells. IND molecules can be divided into groups on the basis of distance from the protein surface and on the basis of hydrogen bonds, either to other IND molecules or to the protein. (b) Diffusivity of IND molecules in different drug shells from the surface of BLG (dry system, 87% drug fraction). Black columns show data for all molecules without any filtering; pink shows the diffusivity for all IND molecules except those bonded to proteins in the first and second generations. Turquoise columns show the diffusivity of IND molecules without hydrogen bonds for more than half of the time throughout the production stage. The dashed line demonstrates the average mobility of all IND molecules in the system. (c) Diffusivity of IND with and without hydrogen bonds (dry system, 87% drug fraction). 50% denotes half of the time of the analyzed timeframe. (d) Mobility of bonded and nonbonded IND at different distances from the surface of the protein, in four systems: 75% IND 25% BLG dry, 87% IND 13% BLG dry, 72% API 22% BLG 5% water, and 83% IND 12% BLG 5% water. All values are higher for the system with the highest drug loading, compared to the second highest. In the dry system with the maximum drug loading, the first generation of hydrogen-bonded APIs remains barely mobile, whereas, under humid conditions, mobility increases almost fivefold. Colors in subpanels (b–d) correspond to different groups and do not stand for the same sets of molecules.

Next, we wanted to test the possible correlation of molecule diffusivity in the presence of hydrogen bonds. The IND molecules were divided into overlapping groups based on two criteria: the distance from the protein (at 4 Å intervals)—to drug shells, and the presence of hydrogen bonds (see Figure 7a–d). We were specifically interested in the IND hydrogen bonded to BLG (called first-generation hydrogen bonds for simplicity) and IND bonded to the former ones (second-generation hydrogen bonds). We then measured the average diffusivity values of the molecules in these groups, as well as for all IND with any hydrogen bonds and for all IND without exclusions.

As was expected, when all IND molecules were considered, diffusivity gradually increased as the distance from the BLG surface increased (Figure 7b, black columns). Once the first and second generations of IND hydrogen bonded to BLG were excluded, the diffusivity of the remaining APIs was found to be slightly greater in all layers (Figure 7b, pink columns). Interestingly, when all of the hydrogen-bonded IND molecules were excluded, the average diffusivity of the APIs became 2–2.5 times higher than the average for all drug molecules (see Figure 7b, turquoise columns, and Figure 7c). This suggests one of two things. Possibly, hydrogen-bond networks not involving BLG also significantly reduce mobility. However, this would contradict the high diffusivity of the pure amorphous drug (Figure 4a). Alternatively, the network is connected to BLG, but for more than two generations. Similar trends were observed for the four other systems: humid and dry, 72–75 and 83–87% drug fractions (see Figure 7d). The humid system had even a lower average diffusivity at the highest of the drug loadings. This might be caused by the higher occupancy of the hydrogen bonds via water molecules that bridge with other water and IND molecules. Lower drug loadings were not studied due to the absence of high-order distances from BLG and high-order hydrogen-bond generations.

In summary, the analysis of hydrogen bonds and diffusivity shows that the IND in the first two drug shells of BLG and the ones bonded to other IND molecules have a noticeably lower diffusivity than the rest of the API molecules. Another important observation is that not all molecules from the FDS have low mobility values. Therefore, it is not exclusively the higher value of local viscosity that determines the stabilization of the APIs at high drug loadings. Thus, we propose that it is the hydrogen-bond networks that stabilize a big portion of the IND mass around the BLGs, in particular with increasing drug loading.

#### 3.2.2 Amino Acids Forming the Hydrogen Bonds

**Figure 8:**
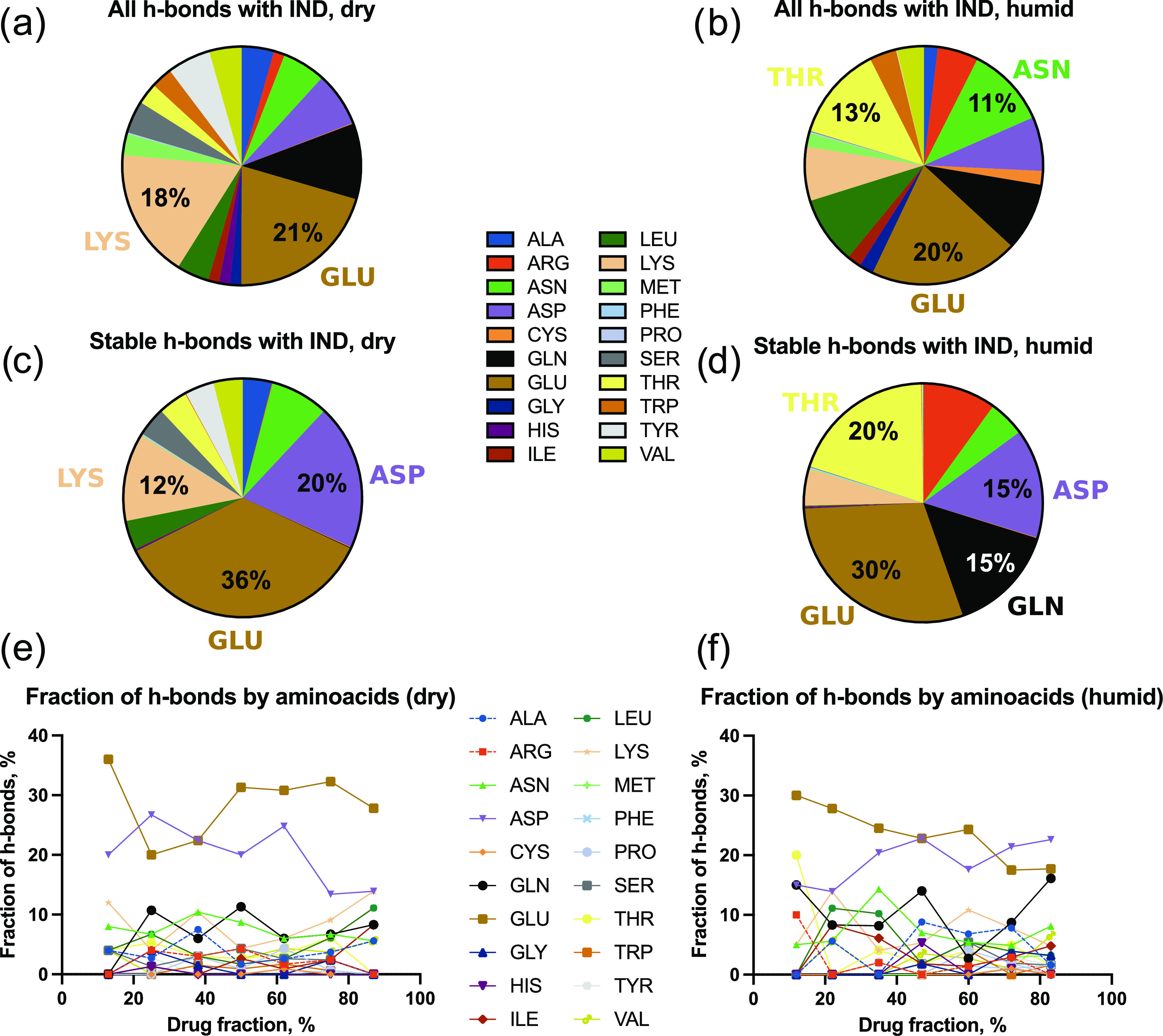
Pie charts of all (a, b) and only stable (c, d) hydrogen bonds (h-bonds) formed between indomethacin (IND) and β-lactoglobulin (BLG), sorted alphabetically and clockwise by amino acids. The pie charts are chosen for 13 and 12% drug fractions for stable hydrogen bonds in dry and humid systems correspondingly. The values are only specified for the major contributions (>10% of the entire number of hydrogen bonds). (e, f) Fractions of the hydrogen bonds formed by specific amino acids as a function of drug fraction. Left panels (a, c, e) represent the dry systems and right panels (b, d, f) represent the humid systems.

It is also of interest to investigate which amino acids form hydrogen bonds (h-bonds) with indomethacin. Hence, the IND-BLG hydrogen bonds from the MD simulations were sorted and ranked (Figure 8). In dry systems, most of the hydrogen bonds were contributed by glutamic acid (GLU), lysine (LYS), glutamine (GLN), and aspartic acid (ASP) (Figure 8a,c). Nevertheless, if only the stable hydrogen bonds are considered, GLU and ASP contribute most of them, whereas GLN is not even represented (Figure 8c,d). In the presence of water, a relatively even distribution between multiple amino acids is seen, but it is GLU, THR (threonine), ASP, and GLN that form stable hydrogen bonds. Irrespective of the drug loading, most of the stable hydrogen bonds were formed by GLU and ASP (Figure 8e,f).

#### 3.2.3 Combination of the First Drug Shell Mobility and Hydrogen-Bond Networks

We conclude that both reduced mobility of the FDS and hydrogen-bond networks are important factors in drug stabilization. The former dominates and is sufficient at the lower drug loadings (up to 30–40%); at these concentrations, IND molecules are located at the surface of BLG and surrounded by a BLG network, and hence, cannot move freely within the ASD (due to high energy penalties associated with such motions). On the other hand, when the mass of the drug is sufficient for formation of drug-rich regions, the mobility of the molecules increases locally, as some of the IND molecules are screened from interactions with BLG. However, as the diffusivity of the drugs in MD simulations and the stability in the experimental setup have shown, ASDs remain stable even at drug loadings higher than 30–40%, i.e., the concentrations at which the drug-rich phase develops. Thus, we propose that at higher drug loadings, other mechanisms contribute to the stabilization of the entire IND mass. At least one of the major mechanisms is then the presence of hydrogen bonds, emanating from the BLG surface (mostly from GLU, ASP, and GLN) through several layers of API molecules.

## 4 Conclusions

In this study, we evaluated the stability of IND-BLG ASDs and investigated the mechanisms of amorphous stabilization. ASDs remained stable for at least 12 months of storage under dry conditions for drug loadings below 80%. Under humid conditions, drug loadings were stable at <40% (ball-milled) and <60% (spray-dried). This could be related to the presence of a drug-rich amorphous phase at loadings of 40–50% and higher as indicated by mDSC. Molecular dynamics enabled an in-depth study of the diffusivity of the molecules in amorphous formulations. Apart from the obvious, higher local viscosity around the BLG, the IND molecules formed far-reaching hydrogen-bond networks throughout the entire drug mass. Hydrogen bonds formed between IND and predominantly the glutamic and aspartic acids in BLG, thereby stabilizing the entire mass at drug fractions up to 75–80%. As observed from the simulations of higher drug loadings, API molecules tended to have a higher diffusion once this threshold was overcome. Interestingly, simulations showed that not the entire FDS around the BLG was fully immobilized. Multiple spots of relatively mobile molecules (compatible with the molecules most distant from BLG) were present within the 0–8 Å range. Small amounts of water did not destabilize the BLG-IND mixture for storage of the drug under dry conditions or moderate humidity. Nevertheless, if exposed to high humidity, the drug molecules could be expelled from BLG at higher drug loadings (>40%).

## Supporting Information Available

Volatile content determination; thermal annealing scheme; XRPD analysis of freshly prepared samples; distributions of the minimal distances between BLG and IND molecules; illustration of the periodic boundary conditions; and mean-square-displacement graph example (PDF)
