# Supplementary material for: Stabilizing Mechanisms of β-Lactoglobulin in Amorphous Solid Dispersions of Indomethacin
Source: Mol Pharm. 2022 Sep 22;19(11):3922–33. doi: 10.1021/acs.molpharmaceut.2c00397 (PMC9644381; doi:10.1021/acs.molpharmaceut.2c00397)
Supplement: Supplementary file 1 — mp2c00397_si_001.pdf [file mp2c00397_si_001.pdf]

# Stabilizing mechanisms of β-lactoglobulin in amorphous solid dispersions of indomethacin

Aleksei Kabedev,[1,#,*] Xuezhi Zhuo,[2,#] Donglei Leng,[3] Vito Foderà,[2] Min Zhao,[4,5] , Per Larsson,[1] Christel A. S. Bergström,[1] Korbinian Löbmann[2,3]

[1]Science for Life Laboratory, Department of Pharmacy, Uppsala University, 75123 Uppsala, Sweden.

[2]Department of Pharmacy, University of Copenhagen, 2100 Copenhagen, Denmark.

[3]Zerion Pharma A/S, Blokken 11, 3460 Birkerød, Denmark.

[4]School of Pharmacy, Queen's University Belfast, Belfast BT9 7BL, UK.

[5]China Medical University – Queen's University Belfast Joint College (CQC), China Medical University, Shenyang 110000, China.

[*]Correspondence: aleksei.kabedev@farmaci.uu.se

[#]The authors were equal in contribution to the work

## Volatile content determination

The volatile content of the ASD samples prepared by ball milling and spray drying was determined on a Discovery Thermogravimetric Analyzer (TGA) using a nitrogen gas purge of 25 mL/min. Approx. 10 mg of each ASDs sample was placed in an open platinum pan and analyzed at a heating rate of 10 °C/min up to 300°C.

Table S1. Volatile content in percent for ball-milled and spray-dried ASDs Ball milling and Spray dry (n =1) at different drug-loadings (DL%)

| DL% wt | Ball milling (%) | Spray drying (%) |
|---|---|---|
| 90 | 2.51 | - |
| 80 | 2.20 | - |
| 70 | 1.86 | 1.19 |
| 60 | 2.94 | 3.87 |
| 50 | 3.41 | 5.02 |
| 40 | 1.50 | 6.46 |
| 30 | 1.40 | 7.47 |
| 20 | 6.04 | 7.37 |
| 10 | 5.11 | 7.64 |

## Thermal annealing scheme

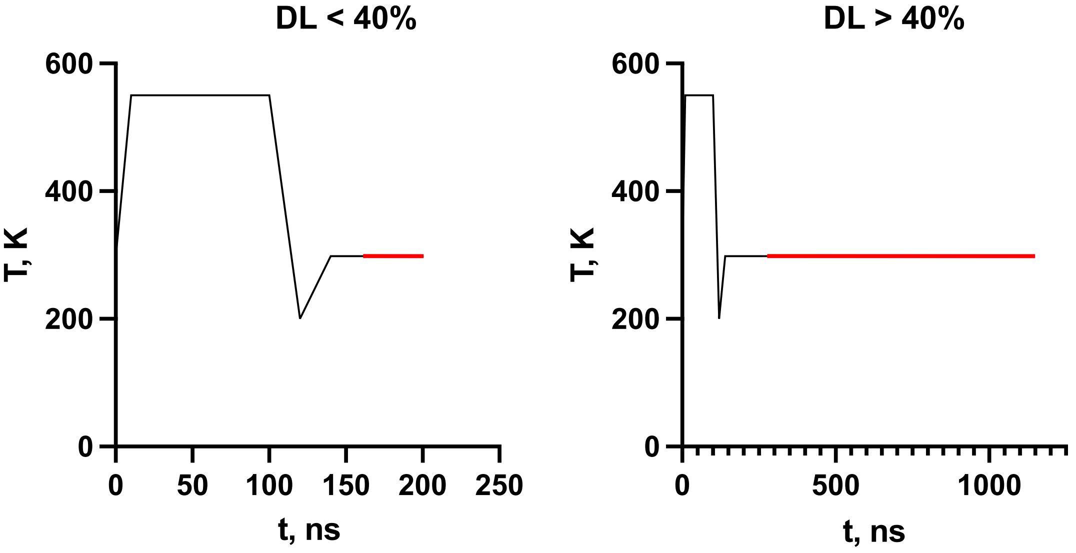


*Figure S1. Temperature profile versus the simulation time shown for systems with high (>40% IND) and low drug loadings on the left and right panels correspondingly. Regions where the measurements were done are schematically depicted with red lines.*

## XRPD analysis of freshly prepared samples

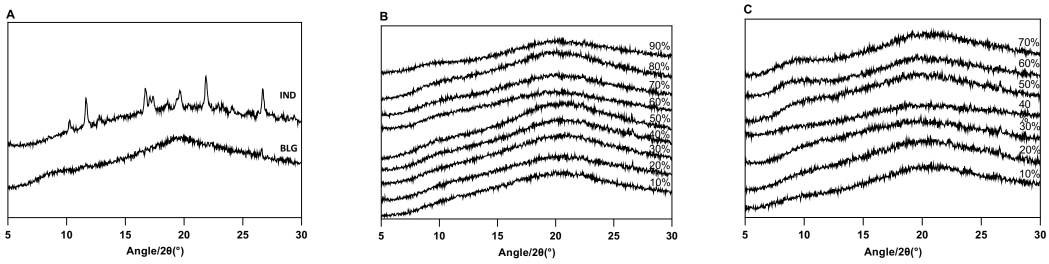


*Figure S2. XRPD diffractograms of freshly prepared (A) bulk IND and bulk BLG, (B) ball-milled samples and (C) spray-dried samples.*

## Distributions of the minimal distances between BLG and IND molecules

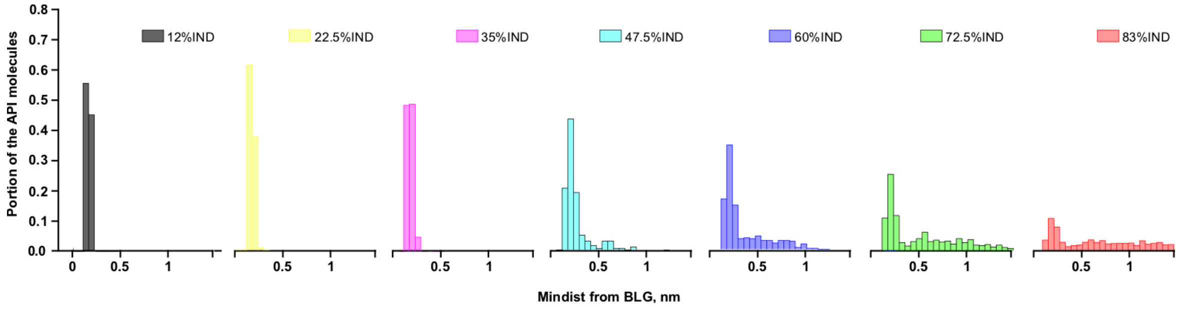


*Figure S3. Histograms of minimal distances between the surface of the protein and IND center-of-mass for humid systems.*

**Illustration of the periodic boundary conditions**

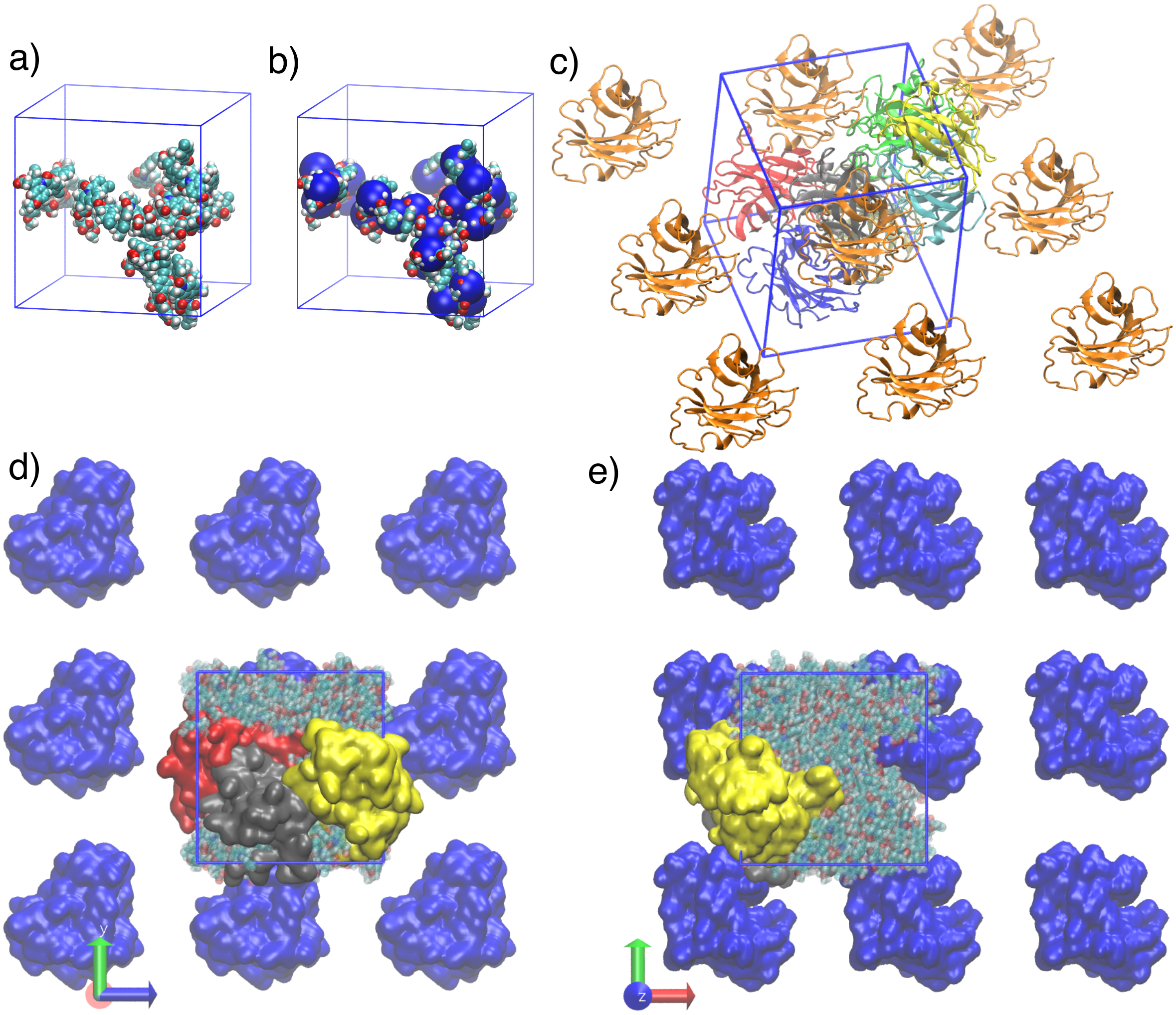


*Figure S4. Clarification of the simulation box dimensions and molecules representation. (a) Depiction scheme for Figure 3 in the main manuscript. (b) Individual IND molecules have been replaced by a single spherical bead (BLG and water molecules are not shown for clarity). Even though some of the blue IND molecules do not seem to be in contact with BLGs, this is not correct, as they are in contact with periodic images of the proteins in one of the dimensions. The calculations were done with an in-house written script for VMD. (c) Box with eight BLGs showing only the proteins (with one presented with 8 periodic images. At the drug loadings below 40% we used eight proteins, so that in every dimension BLGs would not get in contact with their own periodic images. The system shown is 25% IND and 75% BLG. (d and e) For the systems with drug loading higher than 40% the number of APIs was sufficient to screen the interactions of four proteins with themselves over the periodic boundary condition. Thus, the systems were made smaller to reduce computational costs of the simulations. Panels (d) and (e) demonstrate the typical distances between the periodic images of the same protein (blue). The system shown is 50% IND 50% BLG.*

**Mean squared displacement graph example**

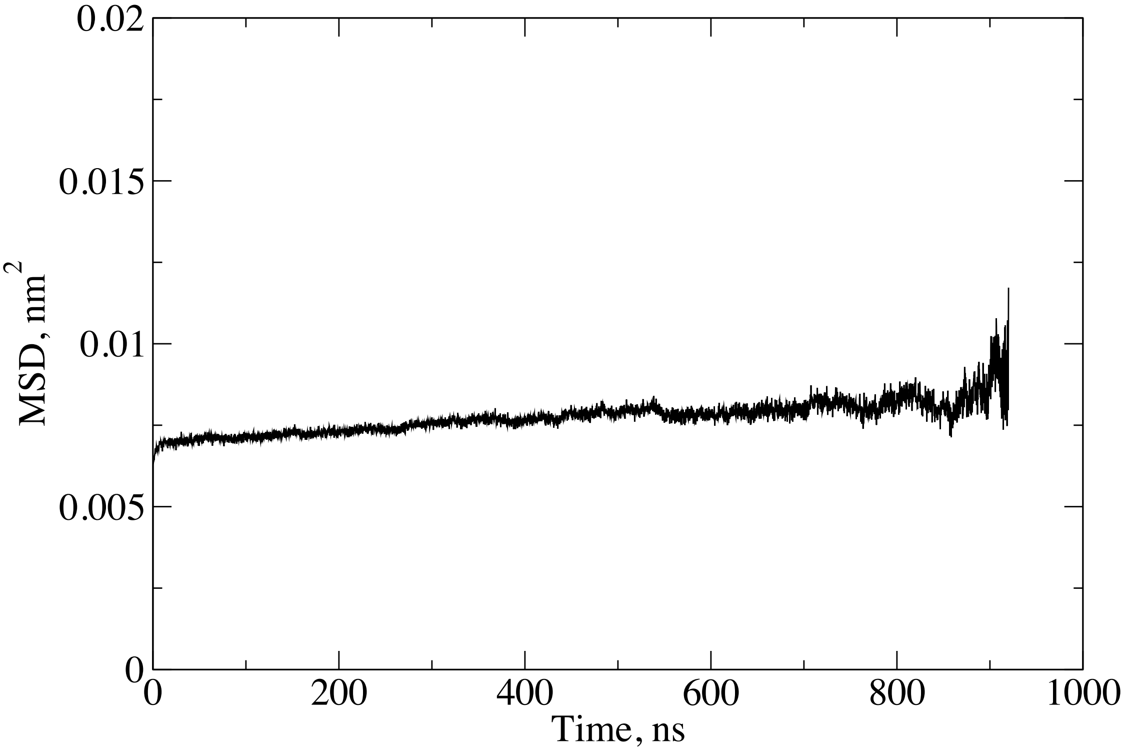


*Figure S5. Example of the mean squared displacement graph for a single IND molecule, used for the calculation of the diffusion coefficient.*
